# Autonomic function testing in spinocerebellar ataxia type 2

**DOI:** 10.1007/s10286-018-0504-4

**Published:** 2018-02-12

**Authors:** Elisabetta Indelicato, Alessandra Fanciulli, Jean Pierre Ndayisaba, Wolfgang Nachbauer, Roberta Granata, Julia Wanschitz, Michaela Wagner, Elke R. Gizewski, Werner Poewe, Gregor K. Wenning, Sylvia Boesch

**Affiliations:** 1 Department of Neurology, Medical University of Innsbruck, Anichstrasse 35, 6020 Innsbruck, Austria; 2 Department of Neuroradiology, Innsbruck Medical University, Innsbruck, Austria; 3 Neuroimaging Research Core Facility, Innsbruck Medical University, Innsbruck, Austria

**Keywords:** Spinocerebellar ataxia type 2, Olivo-ponto-cerebellar atrophy, Cardiovascular autonomic function testing, Orthostatic hypotension, Skin sympathetic reflex

## Abstract

**Purpose:**

To assess whether autonomic failure belongs to the clinical spectrum of spinocerebellar ataxia type 2 (SCA2), an autosomal dominant genetic disorder showing progressive cerebellar and brainstem dysfunction.

**Methods:**

We evaluated cardiovascular autonomic function in 8 patients with SCA2 and 16 age- and gender-matched healthy controls. Other autonomic domains were examined through standardized questionnaires and by testing the skin sympathetic reflex.

**Results:**

Patients with SCA2 showed normal responses to cardiovascular autonomic function tests, with the exception of lower baroreflex sensitivity upon standing compared to controls. In questionnaires, 7 out of 8 patients reported bladder disturbances, while 3 out of 6 tested patients had no skin sympathetic reflex.

**Conclusions:**

We did not observe clinically overt cardiovascular autonomic failure in patients with SCA2. Other autonomic domains (i.e., bladder and sudomotor function) may be affected in the disease.

**Electronic supplementary material:**

The online version of this article (10.1007/s10286-018-0504-4) contains supplementary material, which is available to authorized users.

## Introduction

Spinocerebellar ataxia type 2 (SCA2) is a rare inherited neurodegenerative disorder characterized by chronic progressive cerebellar and brainstem dysfunction [20]. The disorder is caused by a CAG repeat expansion in the *ATXN2* gene and is transmitted in an autosomal dominant manner [20]. SCA2 represents one of the most frequent autosomal dominant ataxias worldwide, and the commonest overall in some geographical spots (e.g., Holguìn province in Cuba) [5]. Disease onset is typically in the 2nd to 4th decades of life with a progressive cerebellar syndrome plus additional features, like slowing of saccade velocity, pyramidal signs, parkinsonism and dementia [16, 20]. Neuropathological studies in SCA2 revealed marked olivo-ponto-cerebellar atrophy, pallor of the substantia nigra and frontal lobe atrophy [11, 20]. Such atrophy pattern highly resembles that of multiple system atrophy (MSA), a sporadic form of olivo-ponto-cerebellar atrophy. SCA2 may even display pontine hyperintensities at MRI, the so-called hot-cross-bun sign, which is considered typical of MSA [2].

Cardiovascular autonomic failure manifests with baroreflex impairment and orthostatic hypotension [i.e., a sustained decrease of systolic blood pressure (BP) ≥ 20 mmHg or diastolic BP ≥ 10 mmHg within 3 min of orthostatic challenge] [10] and has been often described in neurodegenerative disorders with brainstem involvement [1].

Detection of cardiovascular autonomic failure in neurological disorders substantially influences both diagnosis and prognosis [1, 6]. Orthostatic hypotension is indeed a key diagnostic criterion of MSA of cerebellar type (MSA-C). The clinical presentation of MSA-C may overlap that of inherited spinocerebellar ataxias, thus representing a relevant differential diagnosis in the absence of a clear family history [8, 12].

Few clinical studies have previously examined autonomic function in patients with SCA2 [4, 15, 18]. Symptoms of orthostatic intolerance, gastrointestinal, urogenital and sweating disturbances were reported, but most studies were published prior to current consensus criteria for the diagnosis of orthostatic hypotension [10], did not always provide a comparison with age- and gender-matched healthy controls, nor assessed autonomic symptoms through validated questionnaires [4].

Here, we report the findings of a detailed autonomic investigation performed in a genetically confirmed middle European SCA2 cohort versus age- and gender-matched healthy controls.

## Methods

### Study population

Eight patients with genetically confirmed SCA2 and 16 age- and gender-matched healthy controls were enrolled in the present study. The study protocol was approved by the local ethical committee and written informed consent was collected from both patients and controls. All investigations were conducted in accordance with the Declaration of Helsinki.

### Clinical evaluation

Basic clinical–demographic data, as well as results of genetic testing, were collected for all patients. All patients underwent a general and a neurological examination. Disease severity was evaluated with the scale for the assessment and rating of ataxia (SARA) [21]. Laboratory testing was performed to rule out disorders of glucose metabolism, vitamin deficiency or thyroid dysfunction. In the routine work-up, all patients had undergone nerve conduction studies and cerebral MRI. ECG was performed in all patients, while echocardiography was performed in 6 of them.

### Cardiovascular autonomic function assessment

On the day of cardiovascular autonomic function testing, patients and healthy controls were invited not to drink any coffee, tea or taurine-containing beverages and to have their meals at least 2 h before the scheduled examination. All tests were performed between 09:00 a.m. and 12:00 p.m. The test battery included: 10 min supine, 10 min 60° head-up tilt, 5 min supine, 5 min active standing, metronomic deep breathing at 6 cycles/min for 1 min and the Valsalva manoeuvre under continuous non-invasive heart rate and BP monitoring (Task Force^®^ Monitor, CNSystems 2007). Deep-breathing and Valsalva ratio were calculated following standardized methodology [17]. BP contra regulatory behaviour during the late phase II (II_L) and phase IV of the Valsalva manoeuvre were also evaluated as further indices of vascular noradrenergic control [7]. Baroreflex sensitivity in the supine position and during active standing was calculated according to the sequence method [17]. Every patient performed at least the head-up tilt or the active standing.

### Further autonomic evaluations

Autonomic symptoms were investigated by means of two  questionnaires, the SCOPA-AUT and the Orthostatic Hypotension Questionnaire (OHQ) [13, 24]. Sudomotor function was evaluated by testing the skin-sympathetic reflex in 6 patients. Sympathetic skin responses after tibial nerve stimulation were recorded from both feet with surface electrodes. The skin sympathetic reflex was rated as “absent”, if skin responses were missing in both feet.

### Statistical analysis

Statistical analysis was performed with SPSS 24.0. Categorical variables are reported as percentages, while continuous variables are reported as mean and standard deviation or median and interquartile range depending on their distribution, verified by means of the Shapiro–Wilk test. Comparisons between patients and healthy controls were performed by means of unpaired *t* test or ANOVA for repeated measurements (where necessary) for normally distributed variables. The Mann–Whitney *U* test was applied for not normally distributed values. Statistical significance was set at *p* < 0.05.

## Results

### Clinical-demographic data

Our SCA2 cohort consisted of 8 patients (3 women, 5 men) from 5 pedigrees (see Table 1). Median age at examination was 49 years (range 29–56) and mean disease duration was 13 ± 5 years. The average SARA score was 18.3 ± 5.2, which corresponds to moderate ataxia with gait dependent upon devices [23]. Two patients suffered from arterial hypertension; echocardiography was unremarkable in one and showed a mild concentric left ventricular hypertrophy in the other. Two patients were under chronic levothyroxine treatment and had stable thyroid function parameter at the time of evaluation. Four patients had pathological nerve conduction studies, with axonal sensory-motor neuropathy in three of them and pure sensory neuropathy in the other. At MRI, all patients displayed brainstem and cerebellar atrophy and two showed a hot-cross-bun sign (see supplementary Fig. 1).

**Table 1:** Clinical and demographic data for each patient and the whole cohort are shown

	I-1	II-1	III-1	III-2	IV-1	IV-2	IV-3	V-1	Total cohort
Age	48	31	55	50	54	31	29	56	49 (29; 56)
Disease duration	12	6	20	12	15	12	19	7	13 ± 5
CAG repeats (expanded allele)	40	40	40	40	41	46	44	45	42 ± 6
SARA score	25	8,5	19	17,5	18	25,5	18,5	14	18,3 ± 5,2
Cardiovascular comorbidities	No	No	No	Yes	Yes	No	No	No	2/8 (25%)
SCOPA-aut total score	NA^a^	3	3	31	14	3	7	3	3 (3; 31)
Swallowing	-	0	0	2	0	1	0	0	0 (0; 1)
Cardiovascular	-	0	0	3	0	0	0	0	0 (0; 0)
Bladder	-	1	3	7	8	0	2	1	2 (1; 7)
Sexual	-	2	0	0	4	0	0	0	0 (0; 2)
Gastrointestinal	-	0	0	6	2	2	0	1	1 (0; 2)
Sweating	-	0	0	6	0	0	2	1	0 (0; 0)
Thermoregulation	-	0	0	4	0	0	2	0	0 (0; 0)
Pupillomotor	-	0	0	3	0	0	1	0	0 (0; 0)
OHQ	0	0	0	0	0	0	0	0	0
Atrophy pattern at MRI	OPCA	OPCA	OPCA	OPCA	OPCA	OPCA	OPCA	OPCA	8/8 (100%)
Nerve conduction study	Axonal sensory-motor neuropathy	Sensory neuropathy	Normal	Axonal sensory-motor neuropathy	Normal	Normal	Normal	Axonal sensory-motor neuropathy	–
SSR right foot	NA				Absent	Absent	Absent	NA	–
Latency (ms)		2187	1200	1876					
Amplitude (mV)		0.50	0.41	0.20					
SSR left foot	NA				Absent	Absent	Absent	NA	–
Latency (ms)		2243	2043	2164					
Amplitude (mV)		0.81	0.40	0.19					

Pedigrees are indicated by roman numbers (from I to V). Values are reported as median (range), mean ± standard deviation or as percentage, according to their distribution

*OPCA* olivo-ponto-cerebellar atrophy, *SSR* skin sympathetic reflex, *NA* not available

^a^Review of medical records for autonomic symptoms revealed urinary urge and frequency with evidence of overactive bladder detrusor at urodynamic examination in this patient

### Autonomic findings

The median SCOPA-AUT score was 3 (range 3–31). Two outliers, with scores of 14 and 31, were represented by two patients suffering, respectively, from comorbidities unrelated to the underlying neurological disorder (gastric resection and benign prostatic hypertrophy in one case and irritable bowel syndrome in the other). The most frequent complaints were bladder disturbances (7 out of 8 patients), such as increased voiding frequency and/or nocturia (see Table 1). All patients scored zero at the OHQ.

Results from cardiovascular function testing are reported in Table 2. Neither patients nor healthy controls reported symptoms of orthostatic intolerance during cardiovascular autonomic function testing. No patient with SCA2 fulfilled the criteria for orthostatic hypotension, postural orthostatic tachycardia syndrome or experienced syncope during tilt-test examination [10]. In comparison with healthy controls, ANOVA for repeated measurements did not disclose any differences concerning both resting parameters, as well as cardiovascular responses to head-up tilt and active standing. SCA2 patients showed higher, though not significant, supine heart rate (HR) values, with preserved HR increase during orthostatic challenge.

**Table 2:** Cardiovascular autonomic function tests in patients with SCA2 and age- and gender-matched healthy controls

Test	SCA2	Controls	*p*
	*n* = 8, age 49 (31; 55)	*n* = 16, age 49 (30; 55)	
**Head-up tilt**	*n* = 5, age 48 (31; 52)	*n* = 10, age 47 (31; 53)	
*Supine rest*			
Heart rate	71.6 ± 14.5	61.4 ± 8.6	NS
Systolic BP	108 (106.5; 124.5)	112.5 (105.3; 121)	NS
Diastolic BP	75.8 ± 15.5	73.8 ± 7.1	NS
*3′ minute tilt*			
∆ heart rate	+13 ± 7.8	+11.9 ± 8	NS
∆ systolic BP	+4.8 ± 6.1	+9.2 ± 5.9	NS
∆ diastolic BP	+14 (7.5; 15.5)	+11 (10; 13.8)	NS
*10′ minute tilt*			
∆ heart rate	+16.6 ± 7	+12.8 ± 8	NS
∆ systolic BP	+3 (− 0.5; 6.5)	+14 (3; 16.3)	NS
∆ diastolic BP	+3.8 ± 6.2	+10.6 ± 7.8	NS
**Active standing**	*n* = 7, age 50 (31; 55)	*n* = 14, age 50 (31; 55)	
*Supine rest*			
Heart rate	71.4 ± 14.6	62.6 ± 7	NS
Systolic BP	111.9 ± 16.7	116.4 ± 14.7	NS
Diastolic BP	74.4 ± 12.5	70.1 ± 12.5	NS
*3′ minute standing*			
∆ heart rate	+17.3 ± 7.5	+19.5 ± 6.7	NS
∆ systolic BP	+18.7 ± 7.5	+14.9 ± 15	NS
∆ diastolic BP	+17.9 ± 9.5	+22.3 ± 14.4	NS
*5′ minute standing*			
∆ heart rate	+17 ± 7.7	+19.8 ± 6.6	NS
∆ systolic BP	+8 (4.8; 26)	+18.5 (10.8; 25.5)	NS
∆ diastolic BP	+14.3 ± 8	+22.3 ± 14	NS
**Baroreflex sensitivity**	*n* = 5, age 48 (31; 52)	*n* = 10, age 47 (31; 53)	
Supine	11.7 (5.8; 14.9)	16.7 (11.9; 24.5)	NS
Standing	3.7 ± 1.7	9.1 ± 0.6	0.002
**Deep breathing**	*n* = 7, age 50 (31; 55)	*n* = 14, age 50 (31; 55)	
Deep breathing ratio	14.1 ± 10.6	20.6 ± 6.3	NS
**Valsalva manoeuvre**	*n* = 6, age 52 (31; 55)	*n* = 12, age 53 (31; 56)	
Valsalva ratio	1.4 (1.37–2.06)	1.8 (1.62; 1.98)	NS
∆ II_L-II_e mean BP	+12.1 ± 10.3	+9.1 ± 5.6	NS
∆ IV-I mean BP	+7.8 ± 7	+6.5 ± 7.7	NS

Values are represented as mean ± standard deviation or as median (1st quartile; 3rd quartile) according to their distribution

*NS* not significant, *BP* blood pressure

SCA2 patients tended to have lower Valsalva and deep breathing ratios compared to the healthy controls, albeit such differences were not statistically significant. SCA2 patients showed a regular BP overshoot in the phase II-L and IV of the Valsalva manoeuvre. Baroreflex sensitivity in the supine position did not differ between SCA2 patients and controls, whereas SCA2 patients showed a significantly lower baroreflex sensitivity under orthostatic challenge (*p* = 0.002).

Skin sympathetic reflex was absent in 3 out of 6 patients. These patients were members of the same pedigree and had unremarkable nerve conduction studies (see Table 1).

## Discussion

Symptoms of overactive bladder were the most frequent autonomic disturbance reported by patients with SCA 2 in the present study. This finding is in agreement with previous studies, reporting bladder and gastrointestinal symptoms as the most common autonomic features among SCA 2 patients [14, 15, 22].

In our cohort, no patient complained of orthostatic symptoms, which had been reported by 5–13% of SCA 2 patients at SCOPA-AUT questionnaire in previous studies [14, 15, 22]. Here, we used the OHQ, which, being a dedicated questionnaire, may better differentiate true orthostatic symptoms from dizziness or visual disturbances upon position changes due to the underlying cerebellar syndrome.

We observed a largely preserved cardiovascular autonomic function in patients with SCA2. None of the patients showed orthostatic hypotension [10]. Other studies investigating cardiovascular autonomic function in SCA2 patients of Latin American ancestry also did not observe any difference in orthostatic BP behaviour between SCA2 patients and controls [14, 15].

SCA2 patients had a blunted baroreflex sensitivity upon orthostatic challenge with respect to controls. Impaired baroreflex sensitivity was previously reported by Montes-Brown et al. in symptomatic and presymptomatic SCA2 mutation carriers, and possibly reflects the neuropathological involvement of brainstem autonomic nuclei in the disease [14, 15]. However, given the long disease duration and already advanced disease stage of our patients, it appears unlikely for such a finding to evolve into clinically manifest orthostatic hypotension later in the disease course.

Other studies reported cardiovascular autonomic abnormalities in SCA2 patients, but the magnitude of BP fall and/or tachycardia response were not specified [4], or different criteria were applied to diagnose orthostatic hypotension [18]. Another case report on early onset SCA2 pointed out a marked autonomic dysfunction as part of the phenotypic spectrum, but related symptoms were not described [19]. To date, overt orthostatic hypotension has been documented in one patient with SCA2 only [3].

On the basis of the present findings, we conclude that, in spite of the marked brainstem atrophy present in all our patients, overt cardiovascular autonomic failure is not a major feature of SCA2. Nonetheless, involvement of other autonomic domains (i.e., bladder, sudomotor function) may occur [9, 11].

The main shortcoming of this study is represented by the small sample size, which mostly reflects the rarity of the disease in the middle European population. Larger confirmatory studies are required in order to shed light on the possible determinants of selective autonomic disturbances in SCA2.

## Electronic supplementary material

Below is the link to the electronic supplementary material.

Supplementary material 1 (TIFF 1252 kb)
