# Supplementary figures and images for: Autonomic function testing in spinocerebellar ataxia type 2
Source: Clin Auton Res. 2018 Feb 12;28(3):341–6. doi: 10.1007/s10286-018-0504-4 (PMC5995979; doi:10.1007/s10286-018-0504-4)

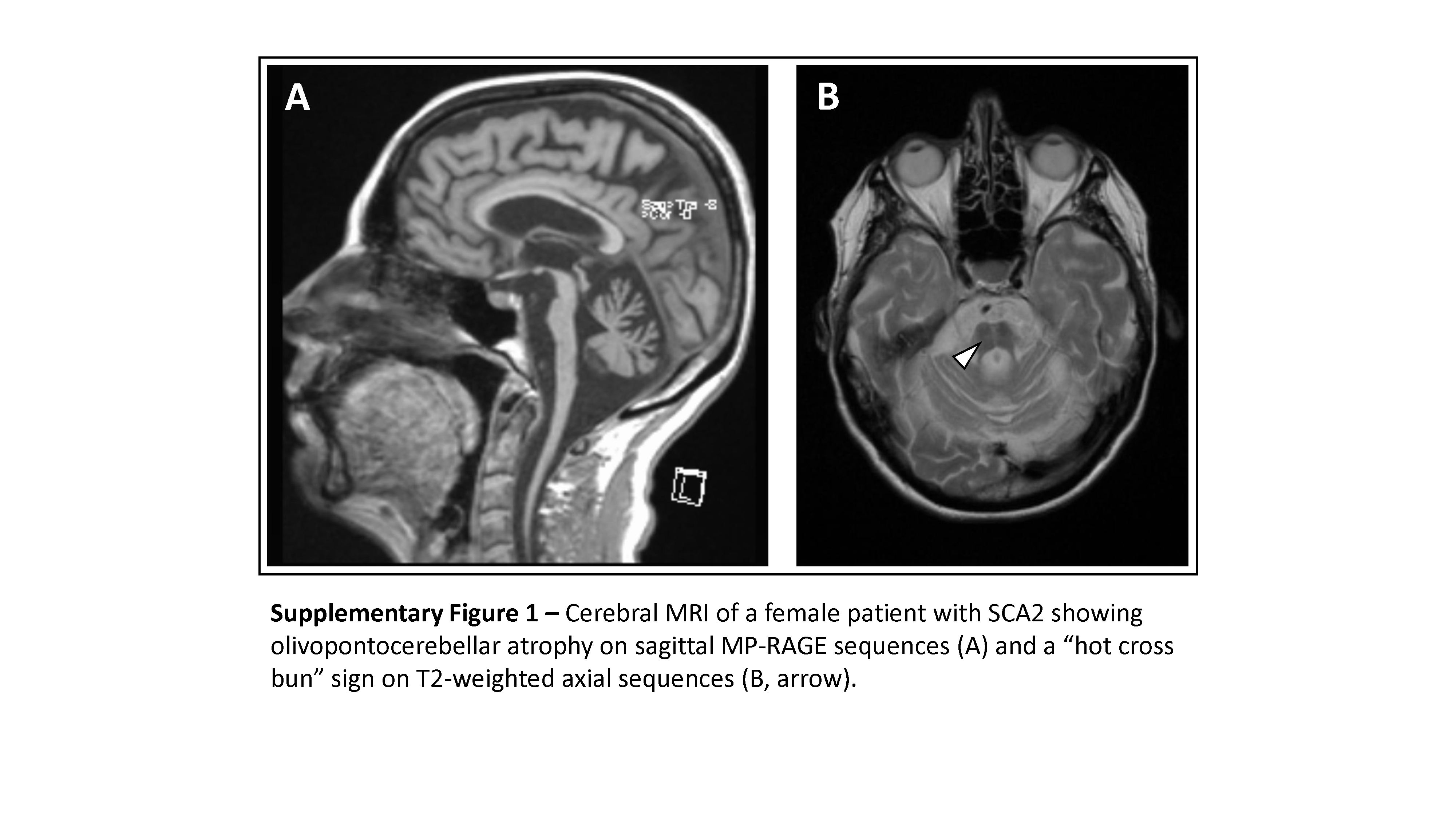

Supplement: Supplementary file 1 — Supplementary material 1 (TIFF 1252 kb) [file 10286_2018_504_MOESM1_ESM.tif]
